# Promotion of Activated Carbon on the Nucleation and Growth Kinetics of Methane Hydrates

**DOI:** 10.3389/fchem.2020.526101

**Published:** 2020-10-06

**Authors:** Guodong Zhang, Xiaoyun Shi, Runcheng Zhang, Kun Chao, Fei Wang

**Affiliations:** ^1^College of Electromechanical Engineering, Shandong Engineering Laboratory for Preparation and Application of High-Performance Carbon-Materials, Qingdao University of Science and Technology, Qingdao, China; ^2^Key Laboratory of Unconventional Oil & Gas Development [China University of Petroleum (East China)], Ministry of Education, Qingdao, China

**Keywords:** methane hydrates, activated carbon, nucleation and growth kinetics, confinement effect, hydrate fibers, two-way convection mechanism

## Abstract

Due to the hybrid effect of physical adsorption and hydration, methane storage capacity in pre-adsorbed water-activated carbon (PW-AC) under hydrate favorable conditions is impressive, and fast nucleation and growth kinetics are also anticipated. Those fantastic natures suggest the PW-AC-based hydrates to be a promising alternative for methane storage and transportation. However, hydrate formation refers to multiscale processes, the nucleation kinetics at molecule scale give rise to macrohydrate formation, and the presence of activated carbon (AC) causes this to be more complicated. Although adequate nucleation sites induced by abundant specific surface area and pore texture were reported to correspond to fast formation kinetics at macroperspective, the micronature behind that is still ambiguous. Here, we evaluated how methane would be adsorbed on PW-AC under hydrate favorable conditions to improve the understanding of hydrate fast nucleation and growth kinetics. Microbulges on AC surface were confirmed to provide numerous nucleation sites, suggesting the contribution of abundant specific surface area of AC to fast hydrate nucleation and growth kinetics. In addition, two-way convection of water and methane molecules in micropores induced by methane physical adsorption further increases gas–liquid contact at molecular scale, which may constitute the nature of confinement effect of nanopore space.

## Introduction

As one of the most promising energy resources, natural gas hydrates gradually get more and more attention, which are stored in permafrost and marine with huge reserves (Sloan, 2003). Unfortunately, such a big piece of cake is not easy to enjoy because of potential tremendous risks, such as seabed geological disasters and greenhouse effect. Hydrates are clathrate structures bonded by water molecules with hydrogen bonds, while guest molecules are captured under demanding conditions (high pressure and low temperature) (Sloan and Koh, 2007). For methane hydrate crystal, a non-stoichiometric composition of 8CH_4_·46H_2_O usually forms with theorical methane storage capacity of ca. 172 m^3^ under standard condition, and this fantastic nature anticipates hydrates to be a promising alternative for the storage and transportation of natural gas, i.e., solid natural gas (SNG), especially in the point of view of cost saving and safety. However, there are technical limits toward industrial application of the SNG technology as a consequence of slow hydrate formation kinetics and low methane storage capacity, so various promoters were employed to overcome such limits, which generally fall into two categories, thermodynamic promoters and kinetic ones (Mech et al., 2016; He et al., 2019).

Hydrate formation is governed by heat and mass transfer, which are improved by interface areas (Clarke and Bishnoi, 2000; Mohebbi et al., 2012), and the presence of solid surfaces can also enhance heterogeneous nucleation (Kvamme et al., 2007; Walsh et al., 2009). Therefore, due to high specific surface area and abundant pore texture, various porous media were reported to significantly enhance hydrate formation kinetics. Typical examples include molecular sieves (Zhou et al., 2005a; Zhong et al., 2016; Liu et al., 2018; Zhao et al., 2018), carbon nanotubes (Park et al., 2010; Zhao et al., 2014), graphite nanoparticles (Zhou et al., 2014; Yu et al., 2016, 2018), MOFs (Mu et al., 2012; Casco et al., 2016), and activated carbon (Zhou et al., 2002, 2005b, 2010; Perrin et al., 2004; Sun et al., 2007; Babu et al., 2013; Borchardt et al., 2018). Impressively, when AC was used, a short or even no induction period was commonly observed (Casco et al., 2015; Borchardt et al., 2016; Cuadrado-Collados et al., 2018), and water can be completely converted to hydrates within 2 h, accompanied with high methane storage capacity (~200 V/V) (Zhang et al., 2020). These fantastic characteristics suggest the possibility of the PW-AC-based SNG technology. However, the fast nucleation and growth mechanisms of methane hydrates loaded by AC have remained, for the most part, unanswered. Keeping this in mind, here we report how methane would be absorbed on PW-AC under hydrate-favorable conditions and evaluate potential micromechanisms behind the fast nucleation and growth kinetics.

## How Methane Would be Adsorbed on PW-AC

**Figure 1:**
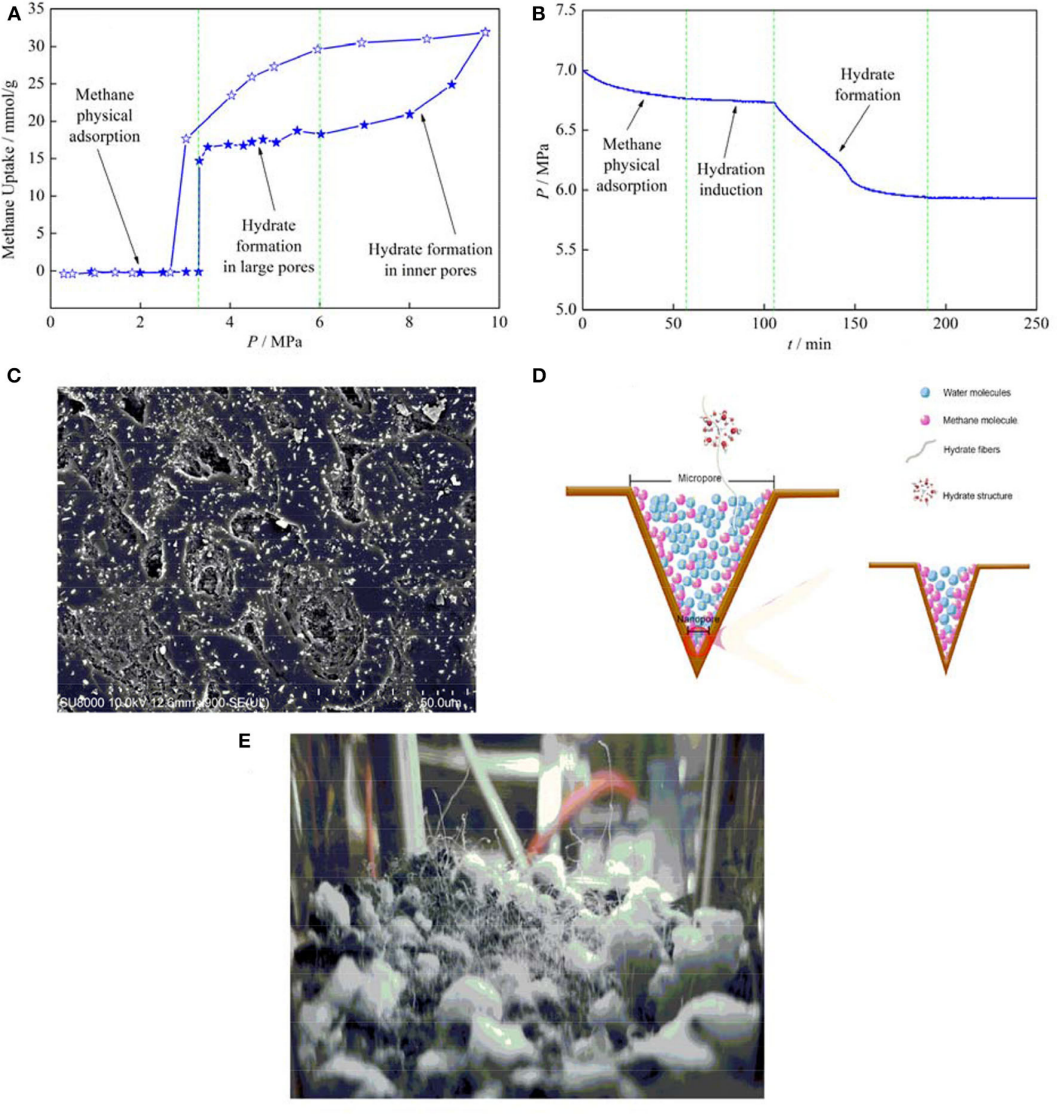
**(A)** Adsorption isotherm, filled pentagrams refer to an adsorption curve, while open ones indicate a desorption curve (Casco et al., 2017) [reproduced with permission from Casco et al. (2017), copyright 2017 Elsevier]. **(B)** Pressure evolution during hydrate formation. **(C)** SEM picture of activated carbon (AC) surface; white substance covering on microbulges refers to NaCl. **(D)** Schematic of two-way convection of water and methane molecules in pore space induced by methane physical adsorption in micropores. **(E)** Hydrate fibers on AC bed.

When PW-AC is used to store methane under hydrate favorable conditions, impressive phenomena are commonly observed, giving rise to hybrid adsorption mechanisms of physical adsorption and hydrate formation. A typical adsorption isotherm (Casco et al., 2017) is shown in Figure 1A, indicating a discrete three-stage adsorption. Apparently, methane physical adsorption in micropores contributes to methane uptake in the first stage, which is almost nil because of steric restriction. However, it significantly depends on the chemical properties of the AC surface, and since strong water molecule–AC surface interaction enhances steric restriction, hydrophobic carbon materials were known as adsorbing more methane (Casco et al., 2019), and some porous materials were even reported to have no influence on methane physical adsorption (Casco et al., 2016). Subsequently, when pressure exceeds a threshold value, i.e., equilibrium pressure, drastic methane uptake occurs that associates with hydrate formation at large pores or pore mouths. It is worth to note that the pressure is higher than that in bulk water because of capillary action (Zhou et al., 2002; Perrin et al., 2004; Liu et al., 2011; Casco et al., 2017), and the extra driving force to overcome capillary action is even higher than 1.5 MPa (see in Table 1). Interestingly, another fast methane consumption stage is observed at higher pressure, which was reported to correspond to hydrate formation in micropores or small mesopores (Casco et al., 2015; Cuadrado-Collados et al., 2018). In addition, it is also worth to note that when hydrate formation kinetics are taken into account by evaluating pressure evolution, new information with more details is observed. We found a three-stage kinetic behavior during hydrate formation as shown in Figure 1B, methane physical adsorption occurs soon after the experimental pressure is reached, followed by a hydration induction period, and eventually hydrates form causing methane quick consumption. This observation constitutes clear evidence on the hybrid effect of methane physical adsorption and hydrate formation on methane storage in PW-AC under hydrate-favorable conditions, so it is not surprising that high methane storage capacity is commonly reported as shown in Table 1.

**Table 1:** Gas storage capacity in preadsorbed water activated carbon (AC).

**Activated carbon**	**Equilibrium condition**	**Experimental condition**	**Gas storage capacity/g·g^**−1**^**	****R_*w*_^†^**	**References**
PP-AC	3.0 MPa, 2°C	10 MPa, 2°C	0.63	4.1	Casco et al., 2015
PP-AC_Ox	3.3 MPa, 2°C	10 MPa, 2°C	0.36	4.1	Casco et al., 2017
C_meso−2_	2.49 MPa, −9°C	8.3 MPa, −9°C	0.34	2.46	Borchardt et al., 2016
Coconut-based AC	4.6 MPa, 1.85°C	10 MPa, 1.85°C	0.32	1.4	Zhou et al., 2002
Corncob-based AC	4.23 MPa, ca. 2°C	9.4 MPa, ca. 1.85°C	0.63	3.35	Liu et al., 2011
CMK_3_1.25	3.27 MPa, ca. 2°C	11.8 MPa, 1.85°C	0.66	3.86	Liu et al., 2006
NC120	3.99 MPa, 4°C	8.5 MPa, 4°C	0.28	1.0	Najibi et al., 2008
NC120	3.49 MPa, 2°C	8 MPa, 2°C	0.35	1.0	Perrin et al., 2004
Picazine	3.7 MPa, 2°C		0.57	ca. 1.0	

† *Mass ratio of water to activated carbon used in hydrate formation experiments*.

Another promising finding for PW-AC-based hydrates is that the presence of AC significantly enhances hydrate nucleation and growth kinetics. Because of the stochastic nature of hydrate nucleation, it is anticipated that the process of qualitative change caused by quantitative change governs hydrate nucleation and growth, so adequate nucleation sites are paramount corresponding to fast formation kinetics at macroperspective. As for activated carbon, abundant specific surface area and pore texture are well-known, which significantly enhance gas–liquid contract and provide tremendous potential nucleation sites, and this constitutes the macromechanisms contributing to fast hydrate nucleation and growth. However, because of complicated surface properties and pore texture, the micronature behind the macromechanisms is still unclear. One of the most appreciated factors was reported to be the chemical properties of AC surface, e.g., surface defect (Pirzadeh and Kusalik, 2013) and hydrophobic or hydrophilic properties (Casco et al., 2017, 2019; Nguyen et al., 2017), which can change the interaction between water molecules and AC surface. Water molecules are known to assemble at the sites which are occupied by surface-deflecting and oxygen-containing functional groups to form clusters, which may provide potential nucleation sites and constitute a hydrate precursor. However, some researchers argued that hydrophobic surface is more efficient, where water molecular clusters occupy the center of inner pores, while methane molecules enrich on the surface (Billemont et al., 2011; Nguyen et al., 2017). In addition, abundant pore texture of AC was also reported to promote hydrate nucleation and growth, i.e., confinement effect, which is considered to remarkably accelerate hydrate nucleation, even within minutes (Casco et al., 2015; Cuadrado-Collados et al., 2018). It is interesting to note that when water and methane molecules exist in a nanopore space, they exhibit a different behavior compared to those out of such confined space under the same conditions. It is well-known that the nanoconfinement effect equals high pressure, the so-called quasi-high-pressure effect (Urita et al., 2011; Fujimori et al., 2013), so solid water was observed in such a pore space because of low activity of water molecules, presenting an ice-like structure, which are thought to enhance hydrate nucleation. Nevertheless, non-freezing water was also reported in a confined nanopore space, which cannot contribute to hydrate formation (Casco et al., 2019; Cuadrado-Callados et al., 2019). Therefore, how the confined pore space influences hydrate nucleation and growth is still unclear due to lack of effective characterization methods at a molecular scale.

Hydrate formation refers to a multiscale process. The nucleation kinetics at a molecule scale give rise to hydrate formation at a macroscale, and the presence of AC causes this to be more complicated. However, the point one should keep in mind is that regardless of what the nature of fast nucleation and growth kinetics of hydrates loaded by AC is, the reason behind that at macroperspective must depend on the increased tremendous potential nucleation sites caused by abundant specific surface area and pore texture. The SEM picture of AC particles is given in Figure 1C. The AC particles were first mixed with a small amount of NaCl solution (unsaturated, only little water remains on AC surfaces) and then quickly frozen to −50°C. Finally, the AC particles were dried by vacuuming, so NaCl would remain on the surface of the AC particles. It is clear from Figure 1C that there are many microbulges (<5 μm) on the surface of AC particles, which are covered by NaCl and show white, indicating that water used to concentrate at these locations, where they would provide nucleation sites for hydrates. Since surface bulges are small and densely distributed on the surface of AC particles, this finding constitutes clear evidence that AC-abundant specific surface area contributes to hydrate fast nucleation and growth kinetic. The last but not the least, it is also worth to note that hydrates usually nucleate during the physical adsorption stage, and we have not observed an induction period in more than 80% of the hydrate formation experiments loaded by AC (Zhang et al., 2020), so it is safe to speculate that there should be a potential relationship between methane physical adsorption and hydrate nucleation. For hydrophobic AC, water molecules commonly occupy the center of a pore space, so the adsorbed methane molecules penetrate into the inner pore through the channel between the pore wall and the water molecules. In addition, methane adsorption in micropores was reported to induce an outward migration of water molecules (Casco et al., 2019), so two-way convection of water and methane molecules in a pore space occurs as shown in Figure 1D, and the enhanced molecular fluidity in a confined pore space significantly increases gas–liquid contact at the molecular scale, which further drastically increases nucleation sites. More details can be found in Zhang et al. (2020). Interestingly, it is clear from Figure 1E that a lot of hydrate fibers form, which grow out from inner pores. This suggests that part of hydrates nucleate in inner pores, and the promotion of confinement effect on hydrate nucleation and growth is also confirmed. To end up, despite the effective characterization methods at the molecular scale required to evaluate hydrate nucleation and growth kinetics loaded by AC, these findings open new perspective to understand the nature behind fast hydrate nucleation and growth kinetics.

## Discussion

The PW-AC-based SNG technology was thought to overcome all the drawbacks of ANG (Zhou et al., 2004), giving rise to higher methane storage capacity and full reversibility of methane, providing this technology is one of the most promising alternatives, but effort is still needed to pay to promote its industrial application. Despite the fast nucleation and growth kinetics commonly observed, one reason behind that is that a tiny amount of AC was usually used, but the scenario changes completely at the industrial scale, where large AC beds retard methane dispersion and decreases gas–liquid contact. Therefore, AC packing density should be carefully considered and optimized (Perrin et al., 2003), which is one of the paramount factors that influence methane storage, transportation, and hydrate formation kinetics. Generally, a dynamic crystallizer may be more efficient to overcome this retardation effect, which may further enhance hydrate formation kinetics. In addition, since the hybrid effect, the total methane storage capacity can be enhanced by improving methane uptake in physical adsorption and hydrate separately, which depends on the physical and chemical properties of AC surface, pore size, and the contents of pre-adsorbed water, etc. As abovementioned, despite hydrophilic surface is considered to correspond to fast hydrate formation kinetics, hydrophobic carbon materials usually associate with higher methane storage capacity, so AC surface modification should be carefully carried out. Furthermore, the contents of pre-adsorbed water can significantly affect the accessibility of inner pores, so an optimal water–AC mass ratio (*R*_*w*_) exists, corresponding to the maximum methane storage capacity and depending on the chemical properties of the AC surface (Celzard and Marêché, 2006; Mahboub et al., 2012; Zhang et al., 2014). Other than that, it is worth to note that *R*_*w*_ also affects hydrate nucleation and growth kinetics, hydrate density, and morphologies, so the optimal *R*_*w*_ should be addressed by taking all those factors into account. The last but not the least, hydrate nucleation and growth kinetics also notably depends on the physical properties of AC, especially the distribution of AC size and pore size. Small AC was reported to correspond to fast nucleation and growth kinetics because of more abundant specific surface area, while larger AC provides adequate interstitial pore space, giving rise to higher methane storage capacity (Siangsai et al., 2015). Moreover, because the lattice size of SI hydrate crystal is ca. 1.2 nm, the minimum pore size for hydrate nucleation was reported to be 1.6 nm (Liu et al., 2011), while the optimal pore size was evaluated to be ca. 25 nm (Borchardt et al., 2016). Ultimately, each kind of AC is unique, so its physical and chemical properties must be evaluated in detail before application, and more efficient AC with appropriate size distribution, pore width, surface properties, etc., should be designed and fabricated to improve hydrate formation kinetics and methane storage capacity (Borchardt et al., 2018).

## Conclusion

Although slow nucleation and growth kinetics limit the industrial application of the SNG technology, the introduction of activated carbon sheds light on it, accompanied with fast hydrate formation kinetics and high methane storage capacity, whereas the mechanisms behind that has not been clearly discussed. The promotion of surface defect, oxygen-containing functional groups, surface physical and chemical properties, and confinement effect have been commonly presented, but they seem not to constitute the essence of fast hydrate formation kinetics. Generally, because of the stochastic nature of hydrate nucleation, it is anticipated that the qualitative change (macro formation) caused by quantitative change (micro multisite nucleation) governs hydrate formation kinetics. Therefore, the increased tremendous potential nucleation sites caused by abundant specific surface area and pore texture should correspond to fast hydrate formation kinetics at macroperspective, and the nature behind them may be numerous microbulges on the surface of AC particles and two-way convection of methane/water molecules induced by methane physical adsorption in micropores.

## Author Contributions

FW prepared the manuscript. GZ wrote the manuscript. XS, RZ, and KC reviewed the manuscript and provided comments, suggestions, and edits. All authors contributed to the article and approved the submitted version.

## Conflict of Interest

The authors declare that the research was conducted in the absence of any commercial or financial relationships that could be construed as a potential conflict of interest.
